# Beyond the “B”: a new concept of the surgical staple enabling miniature staplers

**DOI:** 10.1007/s00464-015-4125-x

**Published:** 2015-03-21

**Authors:** Stefanos Demertzis, Oligca Beslac, Daniel Mettler, Daniel Zalokar, Taylor Spangler, Bernard Hausen, Lee Swanstrom

**Affiliations:** 1 Cardiocentro Ticino & University of Bern, Lugano, Switzerland; 2 Experimental Surgery Institute, University of Bern, Bern, Switzerland; 3 VDx Veterinary Diagnostics, Davis, CA USA; 4 Cardica Inc, Redwood City, CA USA; 5 The Oregon Clinic, Portland, OR USA

**Keywords:** Staples, Surgical staplers, Anastomosis, Laparoscopy

## Abstract

**Background:**

Surgical staplers currently all rely on the same staple form—the “B” which necessitates a high delivery profile (12 mm). A novel “D” shape staple allows for an extremely low profile of the applicator. The acute and long-term efficacy of a D-shaped staple (Cardica, Redwood City, CA, USA) was compared to conventional B-form staples (Covidien, Norwalk, CN, USA) in an animal model for intestinal transections and anastomoses.

**Methods:**

Jejunojejunal anastomoses (JJ) were performed via mini-laparotomy in a swine model. White & blue D- and B-shaped staples were studied in three groups (planned survival 14–84 days). Intraoperative assessment included completeness of staple line, hemostasis, and need for intervention. Postoperatively, animals were evaluated for complications. At the time of sacrifice, gross pathological and histological assessments were performed.

**Results:**

Twenty-three animals had 40 anastomoses (23 “D” and 17 “B” staple anastomoses) with no intraoperative mortalities. One “D” staple application required a manual extension of the cut. Acute hemostasis was 100 %. Group 1 (*n* = 5) compared white staples in JJs (D staple *n* = 5; B staple *n* = 5; 14-day survival = 100 %). Group 2 (*n* = 12) compared white staples in JJs (D staple *n* = 12; B staple *n* = 6; 34-day survival = 92 %). One animal died on day 4 for a non-staple related cause. Group 3 (*n* = 6) compared blue staples in JJs (D staple *n* = 6; B staple *n* = 6; 84 day survival = 84 %). One animal died on day 18 due to an obstruction at the B staple JJ caused by stricture. There were no other bleeding, leaks or strictures in any of the groups. Gross pathology and histology were unremarkable in all JJs.

**Conclusions:**

This study showed no difference in intraoperative performance and the chronic healing response in JJs between D- and B-shaped staples. Based on these findings, the D-shaped staple elicits a normal healing response in jejunostomies and offers the possibility of clinical use of this advance in staple design.

The modern surgical stapler is 55 years old this year. It evolved from the very first surgical stapler developed in 1908 by Dr. Hümer Hültl (1868–1940) which had resemblances of a “B”-shaped staple along with staggered rows [11]. Ever since, medical and most non-medical staplers have been designed and iterated around the “B”-shaped staple. Today, staplers are used in virtually all surgical disciplines and have become the gold standard for the transection and anastomosis of tissues. Surgical staplers are absolutely essential instruments for surgeons worldwide [3].

Over the past 20 years, there have been concentrated efforts by the surgical community to further reduce the invasiveness of their surgical procedures. This started with trocar-based laparoscopic and thoracoscopic procedures using cameras and specialized instruments that could be applied through various size access ports. More recently, surgeons have introduced the concepts of single-port surgery, minilaparoscopy, or natural orifice translumenal endoscopic surgery (NOTES), always driven by the desire to reduce the size of the skin incisions and the invasiveness of the procedures [2, 9, 10, 12, 15, 16].

As a result, the majority of surgical instruments are now available with 5- or 8-mm shaft diameters and offer various degrees of articulation. This includes standard surgical instruments such as graspers, scissors, or irrigation and suction devices. It also includes more sophisticated instruments such as energy-based instruments or clip appliers [14]. However, in regards to surgical stapling, until recently, universally applicable instruments have only been available with a 12-mm or larger diameter shaft.

Traditional laparoscopic staplers utilize cartridges containing multiple rows of wire-formed staples. The unformed staples are shaped in a U configuration (Fig. 4). They are available in different sizes to accommodate different tissue thicknesses. The thicker the tissue, the longer the staple tine needs to be. Based on the current designs, this tine length dictates the minimum size of cartridge that can be realized (Fig. 1). These staples are loaded “on end” into individual pockets in the cartridge. In order to deploy the staple a wedge that passes underneath the staple pockets and pushes individual “drivers” or “pushers” upward, the staples advance sequentially out of the cartridge pockets and into the tissue clamped between the cartridge and the anvil (Fig. 1). The tines of the staples then engage with the anvil and buckle into a B shape, which compresses and secures the tissue. With most staplers, the tissue is simultaneously divided with a knife in the center of multiple rows of staples. Because of this design, and with staple tine lengths approximately 3–5 mm, the conventional stapler cannot be reduced to less than 12 mm in overall diameter (Fig. 1).

**Fig. 1:**
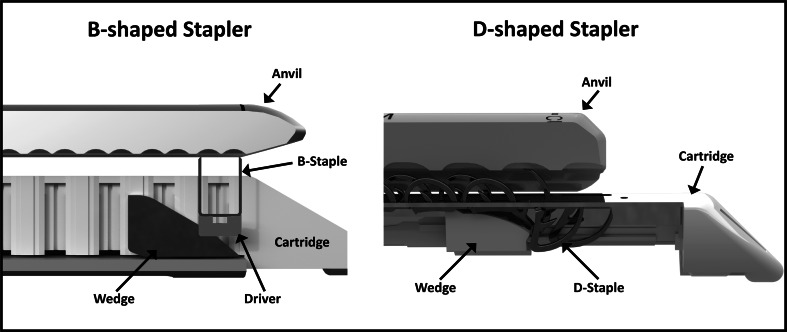
Comparison *B-shaped* versus *D-shaped* end-effector design

The current study evaluates a new type of stapler designed around a fundamentally different staple shape, the D shape. Similar to the conventional B-shaped staples, this new staple is provided with different tine lengths compatible with thin (white cartridge) and medium thickness tissues (blue cartridge).

In contrast to a conventional B-shaped staple, which is formed from constant diameter titanium wire and individually loaded into pockets in the cartridge, the D-shaped staple is placed in multiple rows and is stamped from 316 stainless steel sheet metal as one piece, essentially a strip of staples. The stamping allows for variations in the staple cross section to strengthen sections that are subjected to more force to provide greater resistance to unforming under high tissue loads. In addition, the stainless steel is twice as strong as the titanium wire used to manufacture the B-shaped staples. During deployment, the staples are individually sheared off the strip. The D shape allows the staple to arc through the tissue during deployment and deflect into a closed form by interacting with pockets in the anvil (Fig. 1). This particular design avoids the need for a cartridge housing with pockets for individual staples and “drivers” or “pushers” to translate the force from the wedge onto the staples. Instead the wedge interacts directly with the staple. All of this allows the overall cartridge and anvil diameter to be decreased to 5 mm while accommodating staples capable of securing tissues associated with typical white and blue cartridges.

This study was designed to determine if a 5-mm stapler designed with this new D-shaped staple would be comparable to a conventional B-shaped stapler in a survival model. Outcome included the rate of successful deployments, an assessment of staple forms at the time of deployment, staple-line leakage, and the incidence of staple-line bleeding. Longer term outcome variables included postoperative complications including leaks, bleeding strictures, and death. At the time of sacrifice, a blinded histological assessment of the anastomosis was made.

## Material and methods

The animal studies were performed at the Experimental Surgery Institute of the University of Bern. Animal treatment complied with the 1996 “Guide for the Care and Use of Laboratory Animals” recommended by the U.S. National Institutes of Health. Experimental protocol and animal treatment were approved by the Animal Protection Committee of the Canton of Bern (Switzerland).

Domestic pigs from a local certified farm with a body weight of 20–30 kg were used for the study. The animals were kept at 20–25 °C with daylight and free access to tap water and standard daily food. Before the experiments, the animals were kept fasting overnight with free access to water.

### Anesthesia & positioning

Premedication consisted of an intramuscular injection of ketamin hydrochloride (Ketalar; 20 mg/kg, Pfizer, Karlsruhe, Germany), xylazine hydrochloride (Rompun; 2.0 mg/kg, Bayer Schering AG, Leverkusen, Germany), atropine sulfate (Atropinsulfat; 0.05 mg/kg, Dr. G. Bichsel, Interlaken, Switzerland), and midazolam (Dormicum, Roche Pharma, Switzerland). General anesthesia was induced by intravenous (iv) injection of etomidate (Etomidat-Lipuro; 1 mg/kg, Braun). Anesthesia was maintained by continuous IV administration of fentanyl (Janssen-Cilag AG, Baar, Switzerland) and inhaled isofluran (1.5 %, Baxter AG, Volketswil, Switzerland). Oral intubation was performed (7.5 ET Tube, Portex, Hythe, UK) and the animals were mechanically ventilated (Evita, Dräger, Lübeck, Germany) in a volume-cycled ventilator with a tidal volume of 10 ml/kg, an inspiratory oxygen concentration of 30 % and a positive end-expiratory pressure of 2 cm H_2_O.

### Surgical staplers

Conventional B-shaped transections and anastomosis were performed with a Covidien Endo Gia (Covidien, Norwalk, CN, USA) using 30-mm 6-row white and blue articulating reloads. D-shaped transections and anastomoses were performed with the Cardica MicroCutter stapler (Cardica, Redwood City, CA, USA) using 30-mm 4-row white and blue reloads.

### Animal groups

A total of 23 animals had 40 jejunojejunostomies performed. Three groups of animals were studied to compare white or blue staple loads for both D- and B-shaped staplers. In this animal model, white and blue staples could be used interchangeably. Group 1 consisted of five animals followed for 14 days. Two small bowel anastomoses were performed in each animal using white loads. The anastomoses were created by alternating between the B- shaped and the D-shaped staplers in a random order and the location (proximal distal) and type (D or B) was recorded. Group 2 consisted of 12 animals followed for 34 days. This group included six animals with 2 D-shaped anastomoses in each and six animals with one B- shaped anastomoses. All deployments were performed using white reloads. Group 3 consisted of six animals followed for 84 days. Two small intestinal anastomoses were created in each animal with either the B or D stapler. All deployments were performed using blue reloads.

### Surgical technique

Two jejuno–jejunal (J–J) linear anastomoses were created per pig. A small midline laparotomy was made and a loop of small bowel was exteriorized and transected with a stapler. The two ends were placed in opposing direction and approximated with two stitches placed on the anti-mesenteric borders of the intestine. Enterotomies were then created in both bowel limbs adjacent to each other. Two staple lines were fired through the enterotomies in opposing directions to create a functional end-to-end anastomosis. The enterotomies were then closed with a single running layer of vicryl suture. This was repeated again further distally for a second J–J anastomosis.

### Intra- and postoperative assessment

**Fig. 2:**
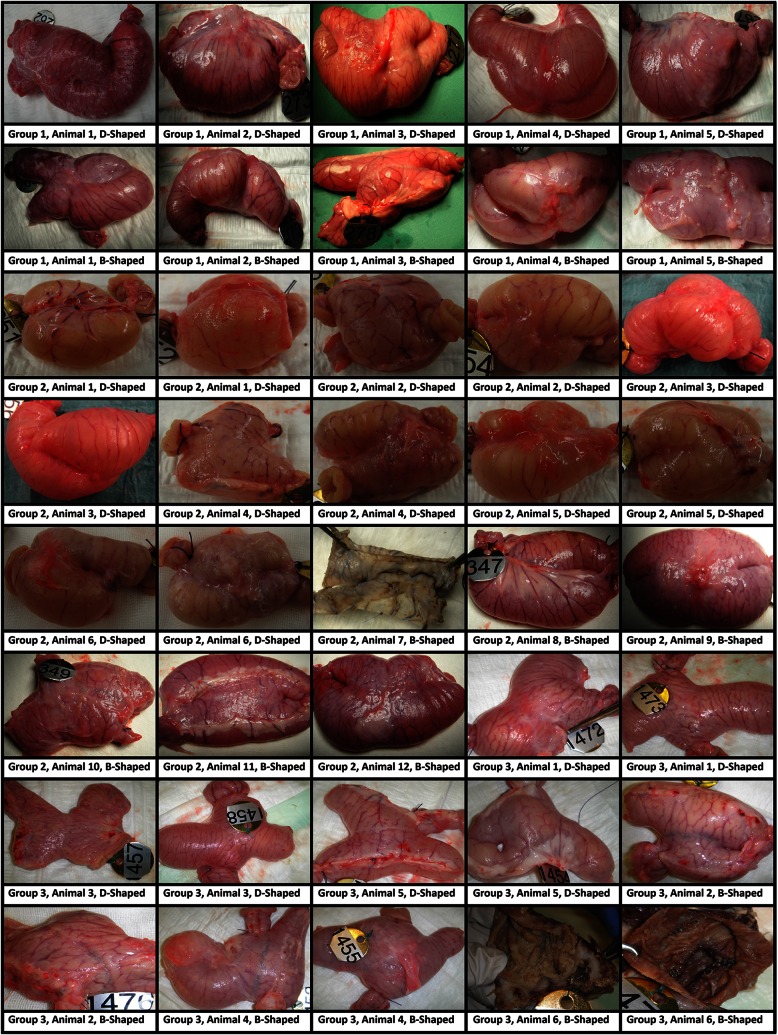
Formalin-inflated small intestinal anastomosis at the time of sacrifice of all animals studied

Intraoperative assessment included the completeness and quality of the staple line, hemostasis, and need for any intervention. Hemostasis was assessed immediately following the deployment and again 2 min later. The procedures were documented by video. Significant bleeding was defined as pulsatile hemorrhage or hemorrhage requiring therapeutic intervention. The number of bleeding locations was documented. The postoperative assessment included length of animal survival and the occurrence of complications events such as staple line leakage, bleeding, or strictures. At the time of sacrifice, the degree of adhesions in the abdomen and the presence of any abnormalities such as strictures or abscess formations were documented. All anastomoses were photographed and then inflated with formalin for gross pathological and histological analyses (Fig. 2). Blood count was performed before surgery and at the time sacrifice. All animals were weighed before surgery and at the time of sacrifice.

### Histology

Individually labeled intestinal anastomosis segments were received at independent histology laboratory (VDx, Davis, CA, USA) for histological preparation and microscopic evaluation. Up to three representative sections along each anastomosis were trimmed, placed in tissue-processing cassettes, routinely processed in graded alcohol, cleared in xylene, embedded in paraffin, cut on a microtome at 4–6 microns, mounted on glass slides, and stained with hematoxylin and eosin and trichrome for microscopic evaluation. During trimming, any staple that was encountered was gently removed from the tissue.

Each sample was evaluated blinded to the treatment method. Semi-quantitative scoring was performed on each sample for the following criteria: inflammation, fibrosis, abscess, necrosis, fistula, serosal reaction around anastomosis, and mucosal loss at the anastomosis. In addition, the predominate type of inflammatory cells that infiltrate around each staple imprint was recorded. Semi-quantitative grading was performed on a scale of 0–4 (0 = absent, 1 = minimal, 2 = mild, 3 = moderate, 4 = severe).

More specific scoring criteria were provided for features of inflammation, fibrosis, serosal reaction/inflammation, and mucosal loss as follows: Inflammation (0 = Absent/1 = Minimal (Patchy, inconsistent mixed inflammation; thin rim around implant)/2 = Mild (Thin consistent rim around implant)/3 = Moderate (Heavier infiltrate beginning to extend away from implant)/4 = Severe (Packed sheets of inflammation extending away from implant); Fibrosis (0 = Absent/1 = Minimal (Averages < 100 microns thick)/2 = Mild (Averages 100–200 microns thick)/3 = Moderate (Averages 200–300 microns thick)/4 = Severe (Averages > 300 microns thick); Serosal Reaction (0 = Absent/1 = Minimal (Very small number of mixed inflammatory cells or edema and expanding serosa)/2 = Mild (More significant inflammation causing mild thickening of serosa)/3 = Moderate (Heavier inflammation with prominent thickening of serosal membrane/4 = Severe (Packed sheets of inflammation and marked thickening of serosal membrane); Mucosal Loss (0 = Absent/1 = Minimal (Focal mucosal defect, not full thickness)/2 = Mild (More extensive area of mucosal loss, may be full thickness, extending away from immediate anastomosis site)/3 = Moderate (Extensive mucosal loss; beginning of ulceration, greater than 1 mm across)/4 = Severe (Significant ulceration into submucosa).

Three tissue samples from each anastomosis were evaluated by the pathologist. The average score from these three readings were used for the statistical analysis. All numeric data were recorded in a FileMaker Pro database (FileMaker 11, FileMaker, Inc., Santa Clara, CA, USA). Additional data, images, videos, reports, and data compilation were collected in a Microsoft OneNote file (Microsoft, Inc., Redmond, WA, USA).

### Statistical analysis

Comparative data from the blinded histological assessment were analyzed using SPSS statistical software (SPPS Version 17, International Business Machines Corp., Armonk, New York). The statistical comparison of this categorical data was performed using the Pearson’s Chi-square test. A *p* value of less than 0.05 was considered significant.

## Results

### Group 1

All five animals underwent surgery without any complications in their immediate and longer-term follow-up. In one of the deployments with the D-shaped stapler, the handle failed during deployment and required use of a replacement handle. All other deployments performed as intended with both the D-shaped and the B-shaped staplers. There was no pulsatile bleeding or bleeding requiring therapeutic intervention. There was no evidence of a staple-line leakage and all staple lines were complete and of good quality at the end of the operation. At the time of sacrifice (po day 14), all small intestinal anastomoses were patent and showed primary healing with minimal adhesions.

### Group 2

The intraoperative course of the 12 animals included in this group was without complications. All deployments were successful and required no therapeutic interventions. There was no bleeding and leakage, and all staple lines looked complete and of good quality at the end of the operation. One animal died prematurely on postoperative day 4. The cause of death was unknown. At necropsy, all anastomoses were patent and transections intact and there was no sign of ischemia or leaks. At the time of sacrifice of the remaining animals in this group (po day 35), all small intestinal anastomoses were patent and showed primary healing with minimal adhesions.

### Group 3

All 6 animals underwent surgery without any major complications. At the time of surgery, all but one deployment in the 6 animals were performed as intended with both the D-shaped and B-shaped staplers. One (1 of 21) deployment performed with the D-shaped stapler had an incomplete cut in the anastomosis that was manually extended. One animal in which all transections and anastomoses had been performed with the B-shaped stapler died prematurely on postoperative day 18. The animal was found to be in extremis and was euthanized. During necropsy in this animal, the two small intestinal anastomoses (B-shaped anastomosis) were found to be adherent to one another and twisted most likely causing an obstruction. At the time of sacrifice of the remaining surviving animals (po day 84), all small intestinal anastomoses were patent and showed primary healing with minimal adhesions.

### Animal weight and blood count analysis

The average animal weights before surgery and at the time of sacrifice are depicted in Table 1.

**Table 1:** Animal weight preoperatively and at the time of sacrifice

Groups		Mean	SD	N
1	Preop	23.5	2.0	5
Survival: 14d	Sacrifice	26.3	0.6	5
	Delta	2.8		
2	Preop	23.5	1.7	12
Survival: 34d	Sacrifice	29.2	3.7	11
	Delta	5.7		
3	Preop	22.8	2.3	6
Survival: 84d	Sacrifice	48.8	7.5	4
	Delta	25.9		

The animal weight gain was commensurated with the length of the follow-up.

The averages from the results of the blood counts performed before surgery and at the time of animal sacrifice are shown in Table 2.

**Table 2:** Blood count preoperatively and at the time of sacrifice

Groups			Hematocrit (l/l) (%)	Erythrocyte S (10e12/l)	Hemglobin (g/i)	Thrombocytes (10e9/l)	Leukocytes (10e9/l)
1 (*N* = 5)	Preop	Mean	30.0	5.7	100.2	398.6	18.3
		SD	1.2	0.4	6.6	143.9	6.5
	Sacrifice	Mean	30.4	5.9	101.6	384.2	18.4
		SD	1.7	0.4	6.4	126.3	3.7
2 (*N* = 12)	Preop	Mean	31.8	6.1	102.3	329.3	15.4
		SD	2.8	0.7	10.3	123.4	4.2
	Sacrifice	Mean	28.5	6.0	99.3	376.4	18.7
		SD	3.0	0.4	10.4	126.0	4.4
3 (*N*−6)	Preop	Mean	30.2	5.8	93.7	359.2	12.9
		SD	1.9	0.5	5.0	87.4	3.3
	Sacrifice	Mean	27.2	5.8	89.2	346.8	14.7
		SD	0.4	0.3	1.9	89.3	3.4

The changes in hematocrit, erythrocyte count, hemoglobin, and thrombocyte count were minor when comparing preoperative blood count with the blood count at the time of sacrifice. The white cell count showed no evidence of major tissue inflammation or infection.

### Histology

**Fig. 3:**
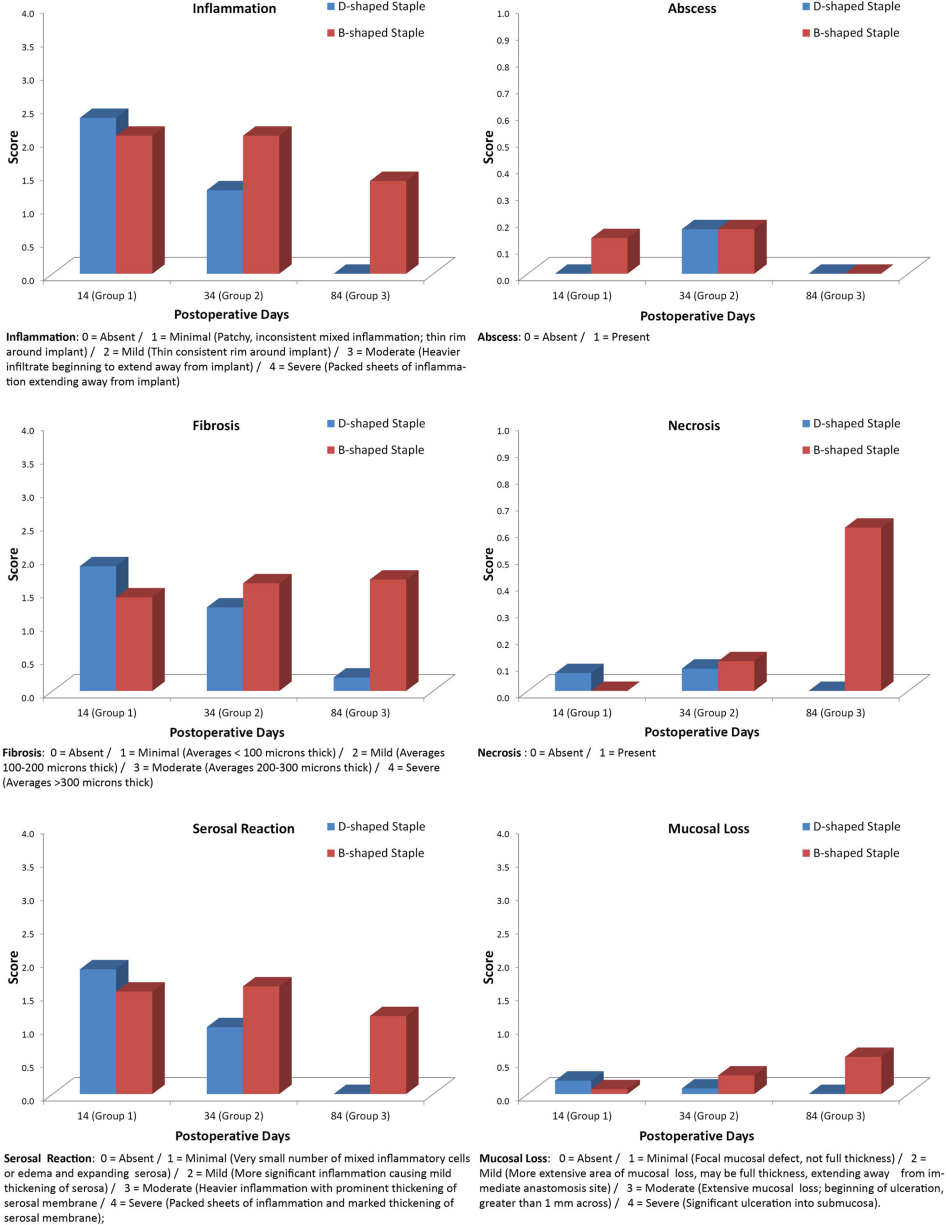
Histological results: comparison of small intestinal anastomosis created using either a *D-shaped* or *B-shaped* staple

Table 3 and Fig. 3 depict the results from the blinded histological assessment of the intestinal anastomosis at the time of sacrifice. The results show that the use of stapling devices is accompanied by mild inflammation, minimal fibrosis, an absence of micro abscesses, fistulas and necrosis, and very little foreign body reaction in tissue samples from small intestinal anastomosis. Overall, there is no meaningful difference in tissue reaction between B- and D- shaped staples used in this study except for a significantly lower grade of infiltration and fibrosis in anastomoses of group 2 D staples when compared to B staples.

**Table 3:** Average histological grades

Group			Infiltration grade	Fibrosis grade	Micro-abscess	Necrosis	Fistula	Serosal reaction grade	Mucosal loss grade
1	D-	Mean	2.3	1.9	0.0	0.2	0.0	1.9	0.2
		*N*	5	5	5	5	5	5	5
		SD	0.6	0.3	0.0	0.4	0.0	0.2	0.3
	B-	Mean	2.1	1.4	0.0	0.0	0.0	1.5	0.1
		*N*	5	5	5	5	5	5	5
		SD	0.6	0.4	0.0	0.0	0.0	0.6	0.1
	Pearson Chi-square		*0.221*	*0.092*	*N/A*	*0.292*	*N/A*	*0.136*	*0.565*
2	D-	Mean	1.3	1.3	0.2	0.1	0.0	1.0	0.1
		*N*	12	12	12	12	12	12	12
		SD	0.5	0.5	0.4	0.3	0.0	0.6	0.3
	B-	Mean	1.7	1.7	0.3	0.2	0.0	1.6	0.3
		*N*	6	6	6	6	6	6	6
		SD	0.4	0.4	0.5	0.4	0.0	0.5	0.5
	Pearson Chi-square		*0.001*	*0.014*	*0.407*	*0.596*	*N/A*	*0.145*	*0.187*
3	D-	Mean	0.0	0.2	0.0	0.0	0.0	0.0	0.0
		*N*	5	5	5	5	5	5	5
		SD	0.0	0.4	0.0	0.0	0.0	0.0	0.0
	B-	Mean	1.3	1.7	0.0	0.5	0.0	1.2	0.6
		*N*	7	6	6	6	6	6	6
		SD	1.0	0.6	0.0	0.8	0.0	1.1	1.0
	Pearson Chi-square		*0.19*	*0.081*	*N/A*	*0.361*	*N/A*	*0.177*	*0.361*

Italicized values represent the *p*-value

## Discussion

The results of this study demonstrate no significant difference in terms of safety and efficacy of D-shaped staples when compared to conventional B -shaped staples. In the 23 animals assessed in this study with a total of 40 small bowel anastomoses, there were no intraoperative or postoperative staple line leaks, stable line bleeds, or strictures. Two of the 23 animals died prematurely. One animal on postoperative day 4 due to an unknown cause with all intestinal anastomoses intact, the other animal on postoperative day 18 with evidence of intestinal obstruction due to adhesions between the two small intestinal anastomoses following placement with B-shaped staples. All other animals were healthy, had gained weight, and had normal blood counts when compared to preoperative values. Blinded histological assessment of the intestinal anastomosis showed evidence of mild inflammation, minimal fibrosis, and absence of micro abscesses, fistulas, or necrosis with very little foreign body reaction. Overall, there was no significant histological difference between the two types of staples used in terms of the chronic healing response they elicited.

**Fig. 4:**
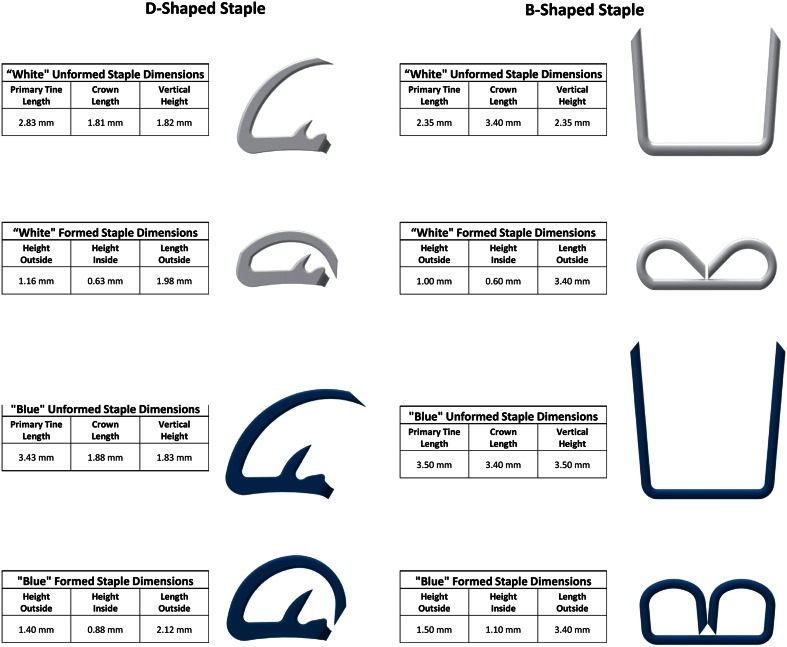
Dimensional comparison of *D-shaped* and *B-shaped* staples

The underlying premise of this study was based on the imminent clinical need for smaller stapling devices. The B-shaped staple, while it has proven itself to be reliable and effective, does not allow a significant reduction in stapler diameter. An alternative staple design was therefore required to enable staplers with significantly smaller shaft. The D-shaped staple used in this study represents one of a number of possible designs that can provide a solution to this engineering challenge. Figure 4 illustrates the configuration of the D staple results in a substantially smaller profile than the B staple while maintaining the same or greater time length: the vertical height of the D staple is 26 % less than the B staple for white cartridges and 50 % less for the blue. At the same time, the tine length is 20 % longer for white and about the same for the blue loads. The fact that the D staples are arranged on their sides in the cartridge results in an even lower profile for the stapler (Fig. 1). In the formed configuration, the inside heights of the two staple designs are practically identical.

In this particular swine animal model, small intestinal anastomoses were selected as the target applications due to the fact that the animals can tolerate small intestinal anastomosis very well and the small intestine can be successfully transected and anastomosed with both white and blue staple sizes. With a maximum intended follow-up period of 3 months, a model was needed where the handling of the animal at the end of the follow-up period was still possible. Also multiple anastomoses can be performed in one animal thereby reducing the overall number of animals required to test the study hypothesis.

**Fig. 5:**
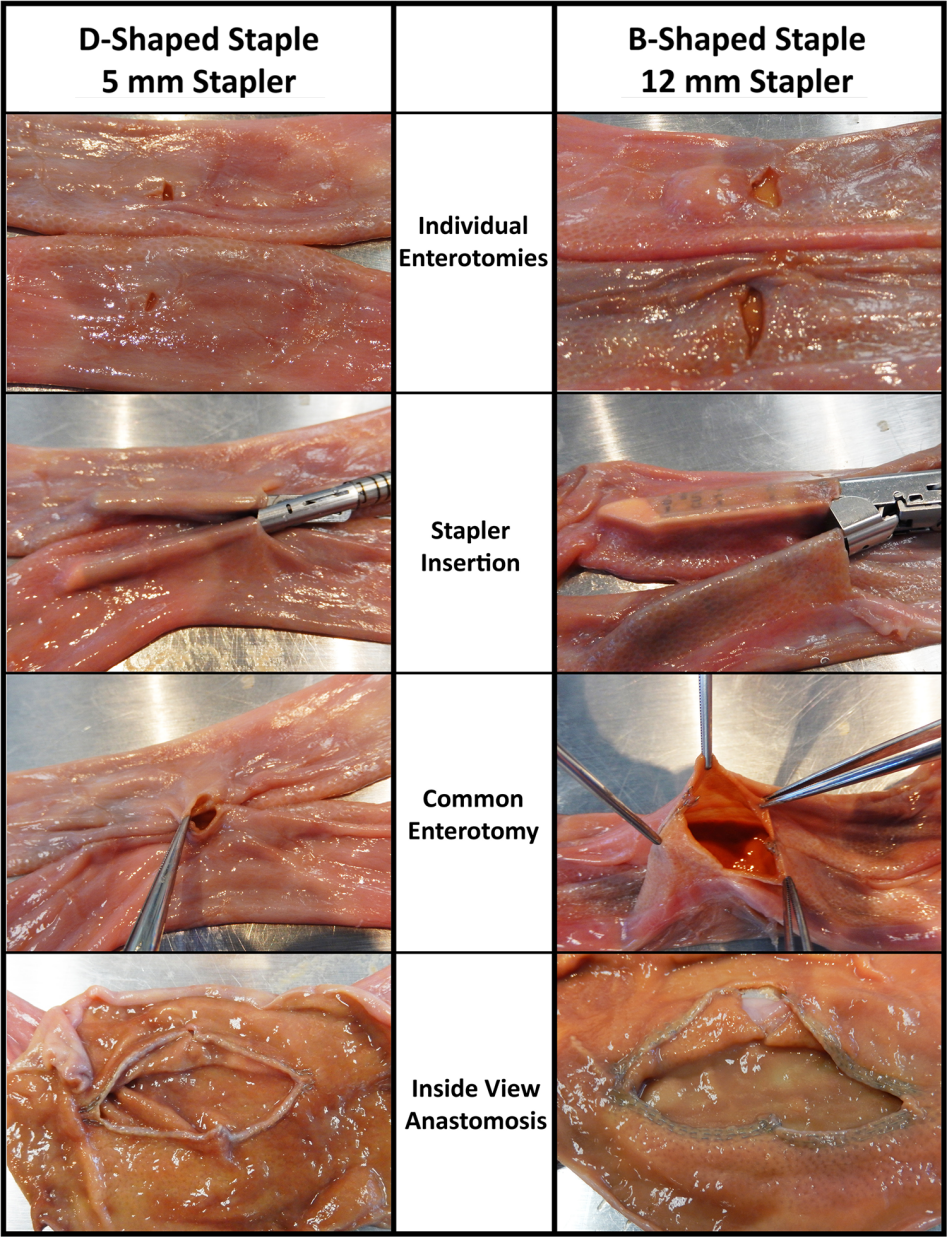
Comparison of common enterotomy and JJ anastomosis between 5- and 12-mm-diameter stapler following one antegrade and one retrograde firing with a 30-mm white staple line using either a *D-shaped* or a *B-shaped* staple

Having a 5-mm diameter laparoscopic staple versus the traditional 12 mm, one offers the potential for many clinical advantages. Obstruction rates at the jejunojejunostomy occur from 1.5 to 4.4 % of the time [1, 8, 7]. In cases where the obstruction was due to a narrowed jejunojejunostomy, the site of narrowing is typically at the closure of the common enterotomy [5, 13, 6]. As illustrated in Fig. 5, a 5-mm stapler allows the traditional side-to-side staple anastomosis to be performed with a very small common enterotomy, typically closed with 1 or 2 sutures. Traditional staples require a 12 mm or larger laparoscopic port for insertion.

The introduction of the D staple has allowed for a significant reduction in the end-effector size for a surgical stapler. This reduced diameter may limit the need for larger 12-mm trocars and their associated higher hernia rates [4]. Further, the surgeon can now use any compatible 5 mm or larger trocar to insert the stapler, significantly increasing flexibility of access. Many tissue structures, especially those in tightly confined spaces, may be more suitable for transection by smaller staplers by reducing the amount of tissue dissection, improving visibility and access. Finally, as shown in the current study, a reduced end-effector can reduce the size of opening created in tissue structures, thereby reducing the amount of subsequent closure required and closure-associated complications.

In conclusion, the current study demonstrates no difference in intraoperative performance when comparing white or blue D- and B-shaped staples for jejunojejunal anastomoses. In this model, the D-shaped staple elicits a normal healing response in jejunostomies as determined at various time points postoperatively. Based on these findings, the D-shaped staple seems safe and effective. Most importantly, this new design allows for a significant reduction in the size of surgical staplers.
